# Inhibition of TLR4 signaling protects mice from sensory and motor dysfunction in an animal model of autoimmune peripheral neuropathy

**DOI:** 10.1186/s12974-021-02126-x

**Published:** 2021-03-22

**Authors:** Oladayo Oladiran, Xiang Qun Shi, Mu Yang, Sylvie Fournier, Ji Zhang

**Affiliations:** 1 The Alan Edwards Centre for Research on Pain, McGill University, 740 Docteur Penfield Ave, Suite 3200C, Montreal, QC H3A0G1 Canada; 2 Department of Microbiology & Immunology, McGill University, 3775 University Street, Montreal, QC H3A 2B4 Canada; 3 Department of Neurology & Neurosurgery, McGill University, Montreal, QC Canada; 4 Faculty of Dentistry, McGill University, Montreal, QC Canada

**Keywords:** Autoimmunity, Inflammation, Demyelination, Macrophages, CD8^+^ T cells, TLR4, DAMPs

## Abstract

**Background:**

While the etiology remains elusive, macrophages and T cells in peripheral nerves are considered as effector cells mediating autoimmune peripheral neuropathy (APN), such as Guillain-Barre syndrome. By recognizing both pathogen-associated molecular patterns (PAMPs) and damage-associated molecular patterns (DAMPs) signals, TLRs play a central role in the initiation of both innate and adaptive immune responses. In this study, we aimed to understand the involvement of TLR4 in the pathogenesis of APN and explore the potential of TLR4 as a drug target for therapeutic use.

**Methods:**

APN was induced by a partial ligation on one of the sciatic nerves in B7.2 (L31) transgenic mice which possess a predisposed inflammatory background. APN pathology and neurological function were evaluated on the other non-injured sciatic nerve.

**Results:**

TLR4 and its endogenous ligand HMGB1 were highly expressed in L31 mice, in circulating immune cells and in peripheral nerves. Enhanced TLR4 signaling was blocked with TAK 242, a selective TLR4 inhibitor, before and after disease onset. Intraperitoneal administration of TAK 242 not only inhibited monocyte, macrophage and CD8^+^ T cell activation, but also reduced the release of pro-inflammatory cytokines. TAK 242 protected mice from severe myelin and axonal loss, resulting in a remarkable improvement in mouse motor and sensory functions. TAK 242 was effective in alleviating the disease in both preventive and reversal paradigms.

**Conclusion:**

The study identified the critical contribution of TLR4-mediated macrophage activation in disease course and provided strong evidence to support TLR4 as a useful drug target for treating inflammatory autoimmune neuropathy.

## Background

Guillain-Barre syndrome (GBS) is an autoimmune disorder characterized by autoreactive leukocyte infiltration into the peripheral nervous system (PNS) leading to demyelination and axonal loss. The incidence of GBS ranges from 1 to 2 cases per 100,000 populations each year [1] and mortality is around 3–7% [2]. Despite intravenous immunoglobulin and plasmapheresis treatments, outcomes are suboptimal. While 60% of patients experience full motor recovery, many of them are left with sensory deficits which do not return to previous functional levels [3]. To date, several hypotheses have been proposed for disease pathogenesis, including the involvement of both humoral and cellular responses associated with autoantibodies, activated macrophages and lymphocytes [4, 5], although the etiology remains unknown.

TLRs are transmembrane receptors expressed by both immune and non-immune cells. They play a central role in the initiation of both innate and adaptive immune responses. These receptors recognize pathogen-associated molecular patterns (PAMPs), such as microbe-specific signatures; and damage-associated molecular patterns (DAMPs), including a wide array of endogenous danger signals derived from damaged cells, such as HMGB1, s100α, heat shock proteins and fibronectin [6, 7]. Increasing evidence shows that excessive activation of TLRs is capable of eliminating immune tolerance by sustained pro-inflammatory and chemokines production, resulting in autoimmune disorders. Increased expression of TLRs 2, 4, and 9, their related signaling molecules (MyD88, TRIF, and NF-kB) have been found in the blood and sciatic nerves of mice/rats suffering experimental allergic neuritis (EAN), a well-established animal model for autoimmune peripheral neuropathy (APN) [8–10]. Similar expression pattern was also reported in the blood samples of GBS patients [9, 11, 12]. However, it remains to be determined whether and how TLR activation can trigger APN and whether inhibition of TLR signaling could be a valid therapeutic strategy to treat GBS patients.

We have previously reported that transgene-derived constitutive expression of the co-stimulator B7.2 (CD86) on antigen-presenting cells of the nervous tissues can cause spontaneous neurological disorders in mice. B7.2 transgenic L31 and L31/CD4^−/−^ mice exhibit both motor and sensory deficits, including weakness and paresis of limbs, numbness to mechanical stimuli and hypersensitivity to thermal stimulation. Many clinical and pathological features observed in these mice resemble to those in GBS patients [13, 14]. Our results also revealed that an injury to a peripheral nerve accelerates the development of APN in *other non-injured nerves* of L31 and L31/CD4^−/−^ mice [15]. The disease in these mice is driven by the synergism between effector/memory CD8^+^ T cells and co-stimulation competent nerve macrophages.

To evaluate the requirement of TLR4 signaling in the initiation and progression of APN, in the current study, we treated L31 mice having ligation on one of the sciatic nerves, with TAK 242, a small molecule compound that selectively inhibits TLR4 signaling by interaction with TLR4 adaptor molecules TIRAP and TRAM [16]. Our results demonstrated that TAK 242 is effective in alleviating APN in both preventive and reversal paradigms. Mice treated with TAK 242 have reduced systemic and nerve inflammation, preserved nervous tissue integrity, and improved functional outcomes. The study identified the critical contribution of TLR4-mediated macrophage activation in disease course and provided strong evidence to support TLR4 as a useful drug target for treating inflammatory autoimmune neuropathy.

## Materials and methods

### Animal model

B7.2 transgenic (L31) mice were generated and interbred as previously described [13, 17]. In these mice, the B7.2 cDNA is under the transcriptional control of MHC class I promoter and Igμ enhancer. Mice spontaneously develop APN between 4 and 6 months. C57BL/6 mice bred and housed in the same facility were used as controls. All data were collected from both male and female mice, since no significant difference was observed between sexes. All procedures were in accordance with the guidelines of the Canadian Council on Animal Care and approved by the animal care committee of McGill University.

### Nerve injury model

Asides the development of APN spontaneously by L31 mice, we previously reported [15] that an injury to one of peripheral nerves accelerated the development of APN in other non-injured nerves in L31 mice. In brief, partial sciatic nerve ligation (PSNL) was performed in L31 mice. Left sciatic nerve was exposed at high thigh level after mice were anesthetized by 3% isoflurane, An 8-0 silk suture was inserted into the nerve with a 3/8 curved, reversed-cutting mini-needle and tightly ligated, one third to one half of the dorsal part of sciatic was trapped in ligature. The wound was closed by a 4-0 skin suture. All data presented in this study are from the L31-PSNL model. All experimental assessments were performed on the right sciatic nerve and right limb.

### Treatment protocol

TAK 242 is a selective inhibitor of TLR4, which binds to the TIR domain of the receptor and disrupts the interaction of TLR4 with its adaptor molecules, thereby inhibiting downstream signaling [16]. The molecule has been used to study the role of TLR4 and its signaling molecules in several inflammatory conditions [6, 18]. The protocol used in this study was adapted from previous *in vivo* experiments with TAK 242 [18]. Briefly, TAK 242 (ApexBio, TX, USA) was dissolved in 10 % dimethyl sulfoxide (DMSO) and further diluted in saline to 1 mg/ml for intraperitoneal injection. To study the preventive effect, mice were treated with either TAK 242 (10 mg/kg b. w.) or vehicle 2 days prior to surgery, then three times a week until day 21 post-PSNL. For the reversal effect, TAK 242 treatment (10 mg/kg b. w.) started at day 17 post-PSNL and was continued for three times a week until day 38 post-PSNL, for a duration of 21 days.

### Behavior test

We assessed mouse neurological functions with different motor and sensory behavioral tests to determine their disability and recovery (*n* = 10–12/group for either preventive or reversal paradigm)

*Clinical scores* as previously described [13] was used to evaluate motor deficits; 0—normal; 1—reduced tonus of tail and/or limp tail; 2—paresis of one hind limb, staying in clasping or outstretching position when lifted by the tail; 3—paresis of both hind limbs, staying in clasping or outstretching position when lifted by the tail; 4—paralysis or splaying of one hind limb; 5—paralysis or splaying of both hind limbs; 6—moribund or death. Data generated in this study was based on the evaluation of contralateral limb only. Signals appearing in the ipsilateral limb were excluded to avoid inference due to PSNL. Therefore, clinical scores that were considered were 0, 1, 3, 5, and 6.

*Grip strength test* was performed to test neuromuscular function. Test was carried out by assessing grasping applied by a mouse using a grip strength meter (Stoelting Co.). Mice were held at the base of the tail and lowered over the grid while ensuring that the torso was parallel with the grid with both the forepaws and hind paws attached to the grid. Mice were then gently pulled back by the tail and the maximal grip strength was recorded in grams. Procedure was repeated twice, and values were averaged. Mice were returned to their home cages for approximately 20 min between each measurement.

*Von Frey test* as previously described was performed to test paw sensitivity to mechanical stimuli [15]. Mice were placed on a metal mesh floor with small Plexiglas cubicles for at least one hour for habituation before testing. Calibrated monofilaments (Stoelting) were applied to the plantar surface of the hind paw, the 50% threshold to withdraw was determined by an average of two tests separated by at least 1 h. An increase in threshold suggests mechanical hyposensitivity.

*Acetone test* was performed to evaluate sensitivity to cold stimulation. A drop of acetone, approx. 25 μL, was applied to the plantar surface of the hind paw, the duration of acetone evoked behaviors (flinching, licking or biting) within 1 min observation was recorded. An increase in the duration of above-mentioned pain like behavior indicates cold hypersensitivity.

### Histological analyses

Axon and myelin structure was visualized using toluidine blue staining on semi-thin nerve cross-sections. Mice (*n* = 5–6/group) were perfused with a fixative solution (0.5 % PFA + 2.5% glutaraldehyde + 0.1 M phosphate buffer) at the end of the experiment. Sciatic nerves were removed and post-fixed in the same fixative overnight. Nerve samples were processed with osmium tetroxide, dehydrated, and embedded in epon kit. Sciatic nerves were sectioned at 0.5 μm using an ultramicrotome (Leica-Reichert) with a diamond knife (Diatome Switzerland). Sections were then stained with toluidine blue for 40 s at 60 °C. Images were obtained by using an Olympus BX51 microscope equipped with a color digital camera. The severity of nerve damage was assessed by measuring demyelinated area over total nerve cross section surface (*n* = 5–6/group). In the area where nerve structure was generally maintained, 100 myelinated axons per section (5–6 nerves/group) were randomly selected to measure myelin and axon area by using ImageJ software (NIH), which allows the assessment of microinjury in sciatic nerves.

### Flow cytometry

Single cell suspension from blood and sciatic nerve of (*n* = 5–6/group) was prepared as previously described [19]. In brief, 50 μl whole blood was collected from sub-mandibular vein of mice and kept in pre-cold Alsevier’s solution (Gibco) to prevent coagulation. After a brief spin down and removal of the supernatant, erythrocytes were lysed by incubating samples with ACK lysing buffer (Thermo Fisher Scientific) at room temperature for 5 min. Approximately, 2-cm-long segments of sciatic was collected following a quick perfusion with 50 μl cold saline. Samples were diced into small pieces and digested by collagenase IV (1.6 mg/ml, Sigma-Aldrich) in 1× HBSS, then passed through a 70-μm cell strainer to obtain the single cell suspension. Following several washes with 1× HBSS, Fc receptors were blocked with 2.4 G2 blocking buffer for 30 min at 4 °C. Samples were then stained with specific fluorochrome-conjugated antibodies for 30 min at 4 °C. Data was acquired with FACS Canto II (BD), and analyzed by using flowjo software. Detailed information of antibodies used in the study is listed in Table 1.

**Table 1:** List of antibodies used for FACS

Antibody	Dilution	Source	Catalog number	Clone
CD8	1:50	eBioscience	17-0081-83, 46-0081-82	53-6.7
CD11b	1:50	BD	552850	M1/70
CD45	1:50	Biolegend	103116	30-F11
CD62L	1:50	eBioscience	11-0621-85	MEL-14
CD86	1:50	eBioscience	12-0862-82	GL1
CD115	1:50	eBioscience	53-1152-82	AFS98
CCR2	1:50	Biolegend	150604	SA203G11
CX3CR1	1:50	Biolegend	149005	SA011F11
F4/80	1:50	eBioscience	45-4801-82	BM8
MHCI	1:50	Biolegend	116517	AF6-88.5
CD44	1:50	Biolegend	103030	1M7
CD43	1:50	Biolegend	121220, 121207	1B11

### Western blot

Mice (*n* = 4/group) were transcardially perfused with 0.9% NaCl. Sciatic nerves (2 cm long) were extracted and homogenized in RIPA buffer (Sigma-Aldrich) containing protease inhibitor (Roche Diagnostics). Homogenization was performed with 0.2 mm glass beads (Sigma-Aldrich) and Precellys 24 tissue homogenizer (Bertin technologies) using three 30 s pulses at 6500 rpm. Protein determination of homogenates was performed by bicinchoninic acid assay (Thermo Fisher Scientific). Equal amount of homogenates (30 μg) were loaded unto 12% polyacrylamide gel. Polyacrylamide gels underwent electrophoresis, and proteins were transferred onto nitrocellulose membranes (Bio-Rad). Successful protein transfer was confirmed with ponceau red (Sigma-Aldrich), and membranes were blocked in 5% non-fat dry milk diluted in 1X TBS-T (Bio-Rad) with 0.1% Tween 20 (Anachemia) for 1 h at room temperature. Membranes were then incubated overnight at 4 S°C with 1:500 rabbit HMGB1 (ABCAM), 1:250 rat TLR4 (Thermo Fisher Scientific) and 1:2000 mouse GAPDH (Millipore). Membranes were then incubated for 2 hours at room temperature with 1:5000 goat anti-mouse (Jackson Immunoresearch) or 1:2000 goat anti-rat (Thermo Fisher Scientific) or 1:2000 donkey anti-rabbit (Millipore) horseradish-conjugated secondary antibodies. Membranes were imaged using super signal west pico chemiluminescence substrate (Thermo Fisher Scientific) on ECL system (Amersham) and quantified using NIH ImageJ.

### Total RNA extraction and RT-PCR

Mice (*n* = 5–6/group) were sacrificed at the end of experiments to harvest sciatic nerve. Total RNA was extracted by using TRIzol reagent (Ambion Life Technologies). In brief, samples were homogenized in TRIzol with 0.2 mm glass beads (Sigma-Aldrich) by using Precellys 24 tissue homogenizer (Bertin technologies) at 6500 rpm for 30 s. Chloroform and isopropanol were then added to remove total protein and genomic DNA, respectively. After washing by 75% ethanol, total RNA was resolved in DEPC treated RNase-free H_2_O. The purity and concentration of RNA was assessed using Nanodrop 2000 (Thermofisher Scientific). One microgram of total RNA was added into each reverse transcription system, which contains superscript IV reverse transcriptase (Invitrogen), Oligo-dt (18mer), and dNTP. Real-time quantitative PCR (qPCR) reactions were processed with a Rotor-Gene Q real-time PCR cycler (Qiagen) using SYBR Green mix from Qiagen (RT2 SYBR Green FAST Mastermix). The levels of target genes were normalized against the housekeeping gene GAPDH and interpreted using the comparative Ct method. qPCR primers were designed based on gene sequence from GeneBank database on NCBI and synthesized by Integrated DNA Technologies. Primer sequences are listed in Table 2.

**Table 2:** List of primers used for RT-PCR

Primers	Forward	Reverse
GAPDH	GTGAAGGTCGGTGTGAAC	AATCTCCACTTTGCCACTG
IL-10	CTATGCTGCCTGCTCTTA	GCTGGTCCTTTGTTTGAAA
IFN-γ	TCCACATCTATGCCACTTGAG	CTGAGACAATGAACGCTACACA
IL-1β	CTATACCTGTCCTGTGTA	GCTCTTGACTTCTATCTTG
IL-6	CTGAAACTTCCAGAGATAC	TTCATGTACTCCAGGTAG
TNF-α	TTCTGTCTACTGAACTTC	CCATAGAACTGATGAG

### Statistical analysis

Data are presented as mean ± SEM and analyzed using the GraphPad Prism software. In general, an unpaired Student’s *t* test was used for single comparisons between groups, and a two-way ANOVA, followed by Bonferroni’s post hoc analysis tests, was used for multiple comparisons. Differences were considered significant at *p* < 0.05.

## Results

### TLR4 and HMGB1 are highly expressed in the blood and nerves of L31 mice

**Fig. 1:**
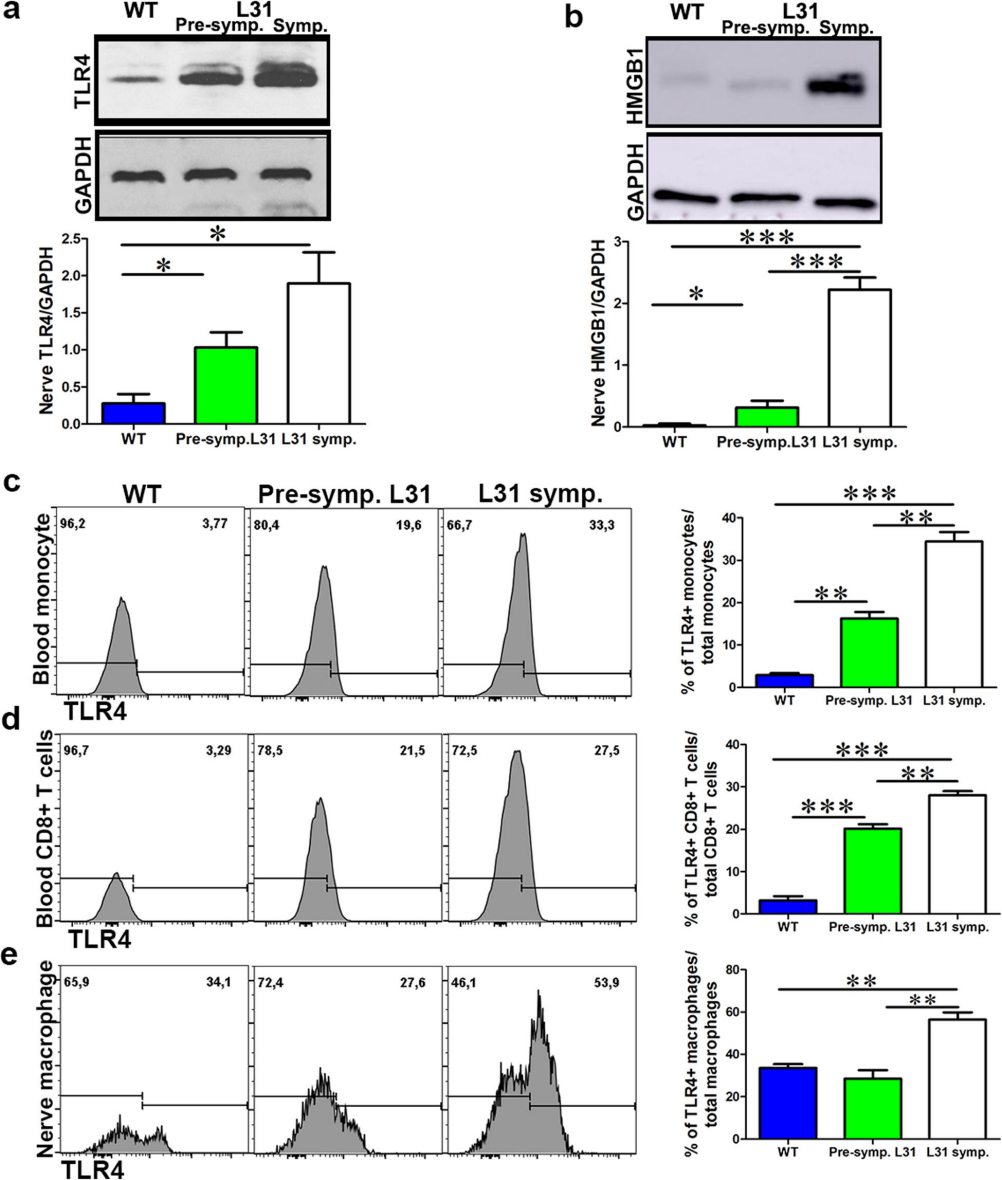
The expression of TLR4 and HMGB1 in the blood and nerve of L31 mice. **a** A representative western blot image showed the expression of TLR4 in the nerve of WT and L31 mice. TLR4 is significantly increased in L31 mice, with the highest increase found in L31 symptomatic mice. **b** A representative western blot image depicted the expression of HMGB1 in L31 mice. HMGB1 expression was increased mainly in the nerve of L31-symptomartic mice. **c** A representative flow cytometry histogram showed monocytes TLR4 expression in the blood. A quantitative analysis showed that TLR4 is robustly expressed in L31 mice with the highest increase found in L31-symptomatic mice. **d** TLR4 is expressed on blood CD8+ T cells in L31 mice with a significant increase in symptomatic mice. Quantification (**c**, **d**) depicted the number of cells per μl blood. **e** TLR4 is expressed on sciatic nerve macrophages in WT and L31 mice. Quantitative analysis showed a significant increase in expression in L31 symptomatic mice. Quantification (**e**) depicted the number of cells per a segment of 2-cm-long sciatic nerve. *n* = 4–5/group, *p < 0.05; **p < 0.01; ***p < 0.001

TLRs are major players in inflammation and in autoimmune neuropathy. To determine the potential involvement of TLR signaling in APN, we examined the expression of TLR4 and the ligand HMGB1 in L31 mice. Western blot analysis revealed that TLR4 expression in sciatic nerves was enhanced, even before the disease onset (Fig. 1a). In parallel, a robust expression of HMGB1, an important endogenous ligand for TLR4, was detected in diseased sciatic nerves of L31 mice (Fig. 1b). Thus, both the endogenous ligand HMGB1 and its receptor TLR4, were upregulated in the sciatic nerves of L31 mice. Further analysis using flow cytometry demonstrated that in addition to the nerves, TLR4 expression was also increased in circulating immune cells. Compared to WT mice, TLR4 expression was upregulated on monocytes (Fig. 1c) and CD8^+^ T cells (Fig. 1d) in the blood of L31 mice, even in pre-symptomatic mice, which was significantly strengthened when they became symptomatic. Similarly, a strong increase of TLR4 expression was detected in immune cells accumulating in the nerves of L31 mice, but mainly in symptomatic L31 mice, with about 60% of Iba1^+^ macrophages in diseased nerves expressed TLR4 (Fig. 1e).

### Blocking TLR4 signaling effectively attenuates sensory and motor deficits in diseased L31 mice

L31 mice spontaneously develop APN at the age of 4–6 months. An injury to peripheral nerve in L31 mice before spontaneous onset accelerates the onset in non-injured nerves [15]. In this study, we performed a PSNL in L31 mice at 2–3 months and evaluated nerve integrity and motor/sensory function of the contralateral limb.

**Fig. 2:**
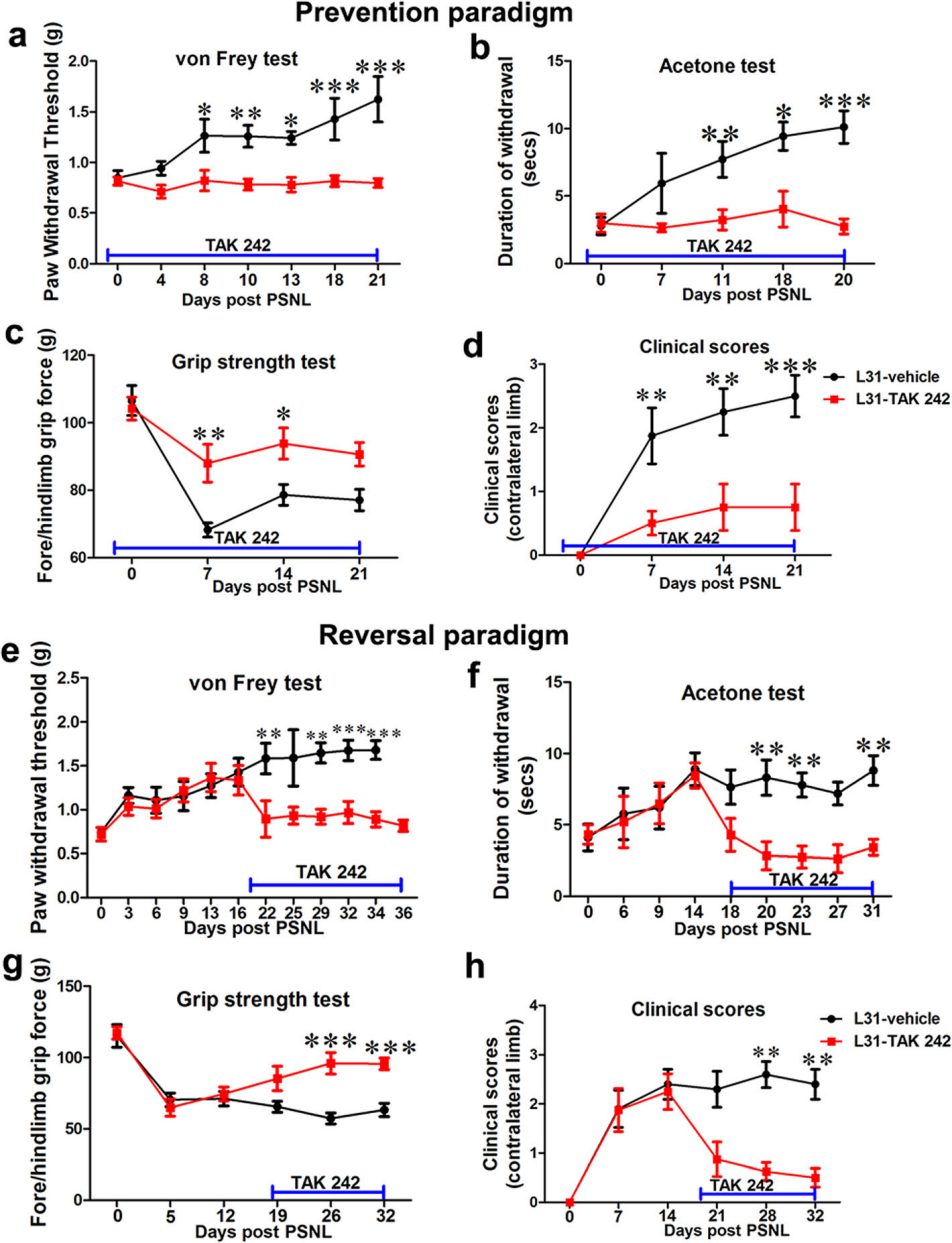
The effect of TLR4 signaling deficiency on sensory and motor deficits/ recovery in L31 mice. In the prevention paradigm (**a**–**d**), **a** compared to mice in the vehicle group, paw withdrawal thresholds assessed using von Frey test maintained to similar level of baseline for mice in treated group, indicating that TAK 242 prevented disease associated numbness/ mechanical hyposensitivity. **b** Thermal sensitivity examined by acetone test was also normal in treated mice. **c** Quantitative data from grip strength showed that muscle strength was greater in treated group compared to vehicle group. **d** Clinical scores showed no appearance of neurological symptoms such as tail weakness and weakness of hind limb in treated group. In the reversal paradigm, treatment started between D17 post-PSNL when sensory and motor deficits were established (**e**–**h**). **e** TAK 242 reversed mechanical hyposensitivity in treated group to pre-PSNL level. **f** Increased paw withdrawal duration in acetone test was equally reversed in treated mice. **g** Quantitative data from grip strength showed that the decreased muscle strength was reversed in treatment group, although not to baseline level. **h** Clinical score showed that the incidence of motor deficits was reduced in treated group. *n* = 10–12/group; *p < 0.05; **p < 0.01; ***p < 0.001

Following PSNL, L31 mice developed abnormal sensory and motor behavior on the contralateral, non-injured paw around 7 days post-injury (Fig. 2), as previously reported [15]. To investigate whether TLR4 signaling is crucial for the initiation and/or progression of disease, we treated L31 mice with a selective TLR4 antagonist, TAK 242, to block enhanced TLR4 signaling, in both prevention and reversal paradigms.

In the prevention group, TAK 242 was administered intraperitoneally at 10 mg/kg, 2 days prior to the nerve injury, then 3 times/week, for 21 days post-PSNL. While mice in the vehicle group started to show persistent mechanical hyposensitivity from day 8 post-PSNL (Fig. 2a), paw withdrawal thresholds remained unchanged in TAK 242 treated mice (Fig. 2a). Similarly, acetone test revealed that cold allodynia observed in the vehicle group was prevented in TAK 242 group (Fig. 2b), suggesting that TLR4 inhibition was able to prevent sensory deficits. Furthermore, blocking TLR4 signaling protected mice from developing motor deficits. Decreased grip strength observed in the vehicle group was ameliorated in TAK 242-treated mice (Fig. 2c). This was further confirmed by the fact that hind limb weakness (increased clinical score) was only detected in the vehicle group starting from day 8 post-PSNL, but not in TAK 242 treated mice (Fig. 2d).

In the reversal group, we investigated the therapeutic effect of TAK 242 administration on disease progression and recovery. TAK 242 administration was initiated at day 17 post-PSNL when both sensory and motor deficits were fully established. The treatment lasted for 21 days. Interestingly, impeding TLR4 signaling also alleviated already established neurological symptoms. TAK 242 completely abolished mechanical hyposensitivity (Fig. 2e) and cold hypersensitivity observed in vehicle-treated-L31 mice (Fig. 2f). TLR4 antagonist also improved mouse motor function in grip strength test (Fig. 2g) and clinical scores (Fig. 2h)

All these behavioral assessments clearly indicated that TLR4 inhibition is sufficient to improve sensory and motor outcomes in a well-established animal model of APN

### Blocking TLR4 signaling decreases monocyte and CD8^+^ T cell activation in the blood of L31 mice

To better understand the effect of inhibiting TLR4 on neurological functions, we first assessed the impact of TAK 242 on systemic inflammation by analyzing the number and the phenotype of monocytes and CD8^+^ T cells in the blood.

**Fig. 3:**
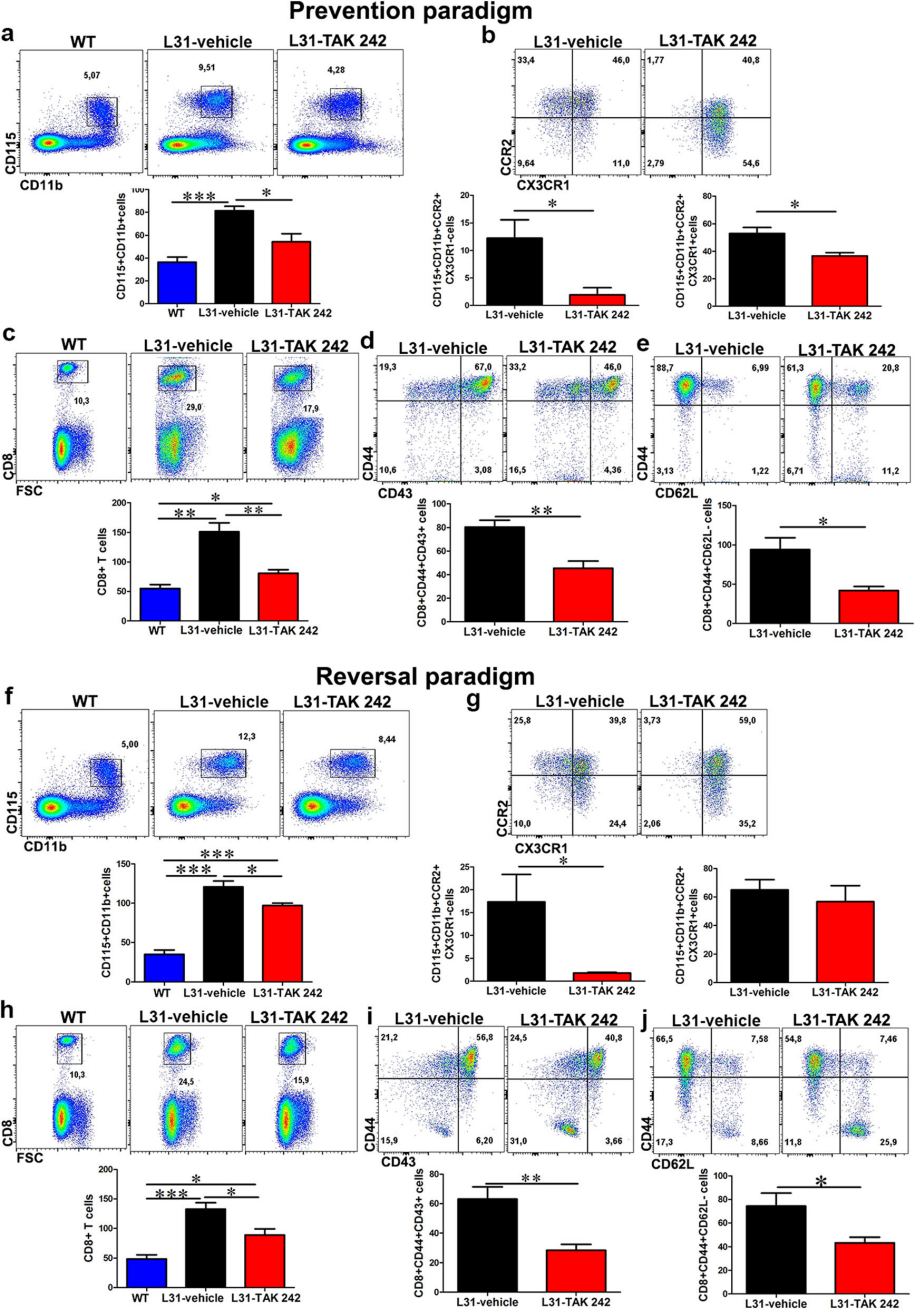
The effect of TLR4 signaling inhibition on monocyte and CD8^+^ T cells activation status in the blood of L31 mice. In the prevention paradigm (**a**–**e**), **a** representative flow cytometry dot plot and quantification analysis showed a decrease in the number of CD115^+^CD11b^+^ monocytes in the blood of treated mice following TLR4 inhibition. **b** CCR2^+^ monocytes (pro-inflammatory) subset which includes CCR2^+^/CX3CR1^−^ and CCR2^+^/CX3CR1^+^ were significantly reduced in treated group. **c** Similarly, the frequency and number of CD8^+^ T cells were reduced in the blood of treated mice. **d** The majority of the reduced CD8^+^ T cells in the treated group were of the activated subset (CD44^+^CD43^+^). **e** The frequency and number of CD8^+^ T cells with effector memory phenotype (CD44^+^CD62L^−^) were significantly reduced in the blood of treated mice. In the reversal paradigm (**f**–**j**), **f** frequency and total number of CD115^+^CD11b^+^ monocytes was significantly reduced in treated group. **g** The inflammatory subset (CCR2^+^CX3CR1^−^) group was significantly reduced in treated mice as shown by representative flow and quantitative data. CCR2^+^/CX3CR1^+^ number appeared similar in both groups. **h** CD8^+^ T cells frequency and absolute number reduced in the blood following TLR4 signaling inhibition. **i** Majority of the reduced CD8^+^ T cells were of the activated (CD44^+^CD43^+^) and **j** effector memory phenotype (CD44^+^CD62L^-^). All quantitative analyses are shown as number of cells per μl blood. *n* = 6/group; *p < 0.05; **p < 0.01; ***p < 0.001

Compared to WT mice, there was a significant increase of circulating CD115^+^CD11b^+^ monocytes in diseased L31 mice (Fig. 3a, f). TAK 242 treatment was able to reduce such increase in both prevention and reversal paradigms (Fig. 3a, f). It is interesting to note that the majority of monocytes in the vehicle group were of the CCR2^+^CX3CR1^-^ and CCR2^+^CX3CR1^+^ inflammatory subsets (Fig. 3b, g). Following inhibition of TLR4 signaling, the CCR2 single positive subset was completely abolished (Fig. 3b, g). CCR2 and CX3CR1 double positive subset was decreased in the prevention but not in the reversal group, which suggests TAK 242 could impact monocyte egress from bone marrow by targeting more specifically CCR2^+^ inflammatory cells. Similarly, we found a significant reduction in the number of CD8^+^ T cells in the blood of TAK 242-treated mice when compared with the vehicle group (Fig. 3c, h), in both treatment paradigms. In addition, TAK 242 also significantly decreased the number of CD44^+^CD43^+^ effector CD8^+^ T cells (Fig. 3d, i) and the number of CD44^+^CD62L^-^ effector/ memory CD8^+^ T cells (Fig. 3e, j) in the blood. These results suggest that blocking TLR4 signaling either prior to or after PSNL can attenuate systemic inflammation by reducing circulating immune cell activation.

### Blocking TLR4 signaling inhibits macrophages and CD8^+^ T cells mediated local inflammation in sciatic nerves of L31 mice

Macrophages and activated CD8^+^ T cells are the effector cells found in peripheral nerves of diseased L31 mice [13, 15]. To further confirm the effect of blocking TLR4 signaling in local inflammation in diseased peripheral nerves, we examined the number, phenotype and function of macrophages and CD8^+^ T cells in sciatic nerves of L31 mice having PSNL, treated or not with TAK 242.

**Fig. 4:**
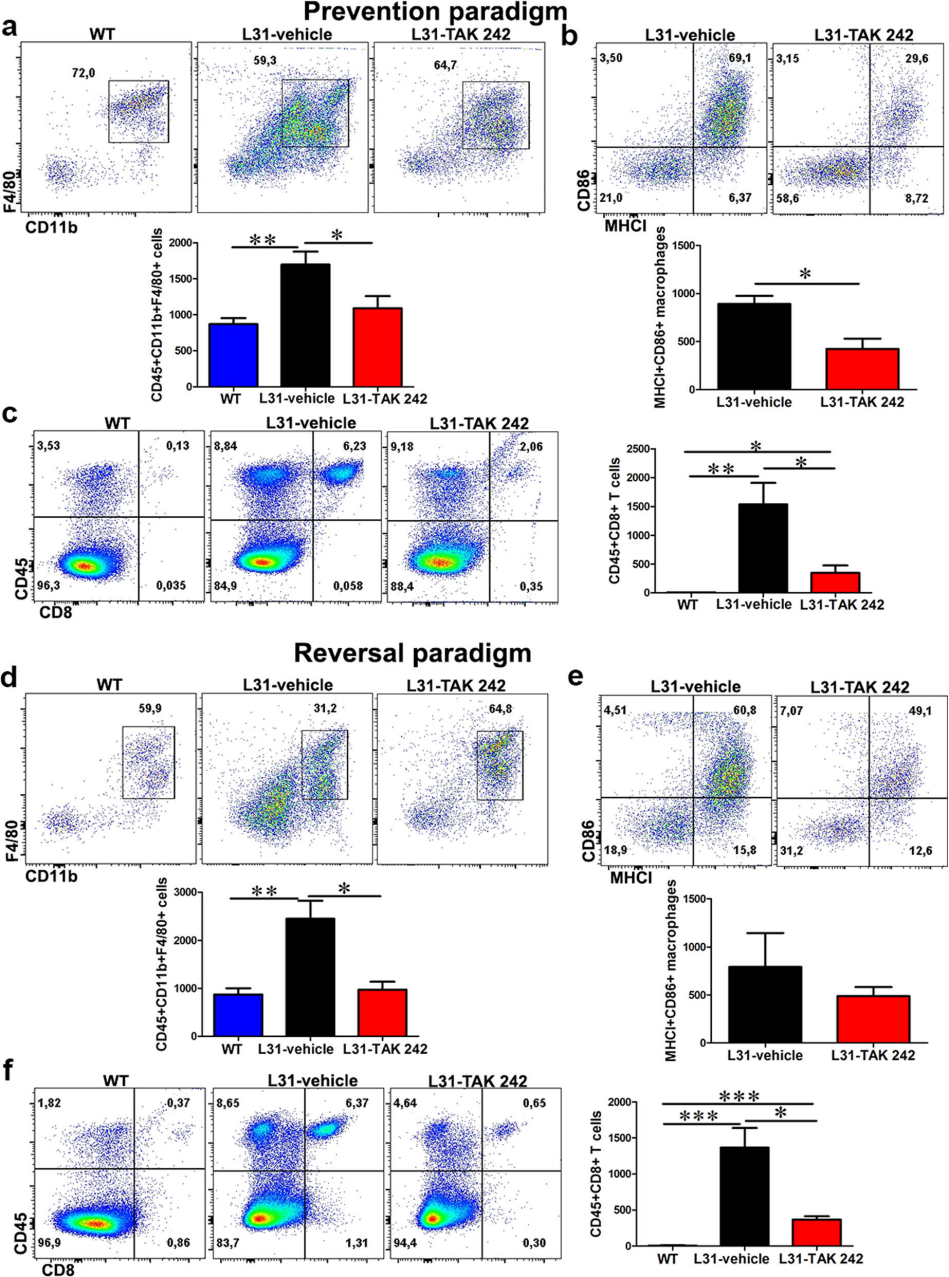
The effect of TLR4 signaling inhibition on macrophage and CD8^+^ T cells number and activation status in the nerve of L31 mice. In the prevention paradigm (**a**–**d**), **a** quantitative analysis from flow cytometry showed a reduced number of macrophages (CD45^+^CD11b^+^F4/80^+^) in treated group at the end of the experiment. **(b)** The number of activated macrophages (MHCI^+^CD86^+^) were significantly reduced in treated group. **c** Frequency and absolute number of infiltrating CD8^+^ T cells were significantly diminished in treated group. In the reversal paradigm (**d**–**f**), **d** quantitative analysis from flow cytometry showed that TAK 242 favored the reduction of nerve macrophages (CD45^+^CD11b^+^F4/80^+^). **e** A quantitative analysis indicated a reduced number of activated macrophages (MHCI^+^CD86^+^). Majority of the reduced macrophages in treated group were those committed to antigen-presenting function (MHCI^+^CD86^+^). **f** A representative flow cytometry dot plot showed a reduced frequency and number of infiltrating CD8^+^ T cells in the sciatic nerve of treated mice. All quantitative analyses are shown as number of cells per a segment of 2-cm-long sciatic nerve. *n* = 6/group; *p < 0.05; **p < 0.01; ***p < 0.001

Quantitative analysis using flow cytometry revealed that TAK 242 significantly reduced the number of activated macrophages and CD8^+^ T cells in the sciatic nerves. In both prevention and reversal paradigms, cells isolated from sciatic nerves were first analyzed for leukocyte common antigen CD45^+^, the identity of macrophages and CD8^+^ T cells were further confirmed using more specific markers. As shown in Fig. 4a, d, in vehicle-treated mice, the number of CD11b^+^F4/80^+^ macrophages reached to 1698 ± 179.7 cells/nerve in the prevention group, and 2547 ± 373.2 cells/nerve in the reversal group, in the contralateral, non-ligated sciatic nerve. TAK 242 treatment was able to significantly reduce these numbers to 839.2 ± 128 cells/nerve in the prevention group and 1040 ± 164.4 cells/nerve in the reversal group (Fig. 4a, d), which reflects the number of resident macrophages in a normal nerve as seen in WT mice (Fig. 4a, d). Indeed, not only reducing the total number of nerve macrophages, TAK 242 decreased more specifically the number of MHCI^+^CD86^+^ macrophages (Fig. 4b, e) which are essential for antigen presentation. As expected, in parallel with the reduced number of macrophages in sciatic nerves, the number CD8^+^ T cells was also significantly reduced in the TAK 242 treated group (Fig. 4c, f). As a whole, blocking TLR4 signaling is effective in reducing infiltration and expansion of immune cells in nervous tissues.

**Fig. 5:**
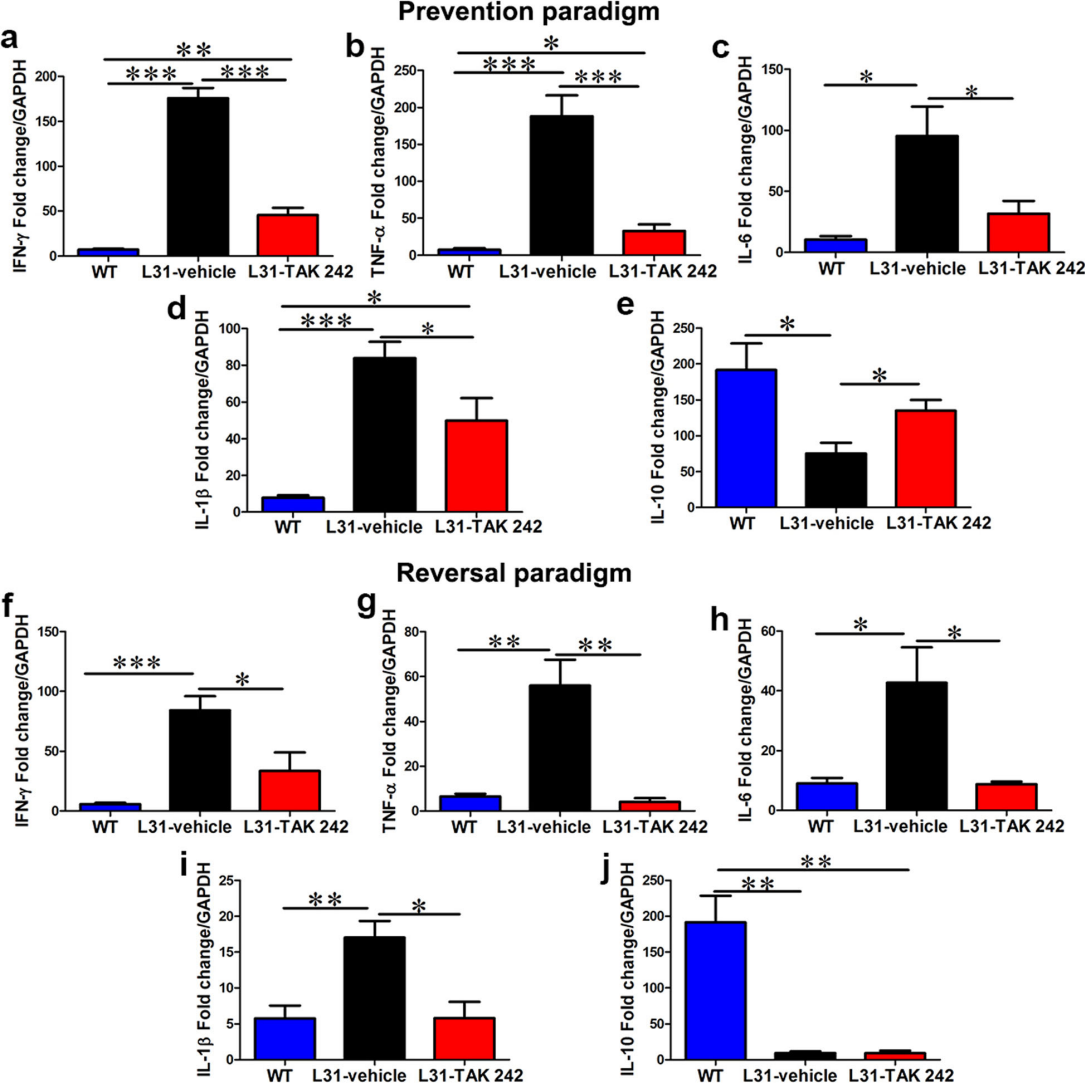
The effect of TLR4 signaling inhibition on cytokine expression in L31 mice. In the prevention paradigm (**a**–**e**), real-time quantitative PCR showed a significantly reduced expression of pro-inflammatory molecules **a** IFN-γ, **b** TNF-α, **c** IL-6, and **d** IL-1β, in the TAk 242-treated group. **e** The mRNA expression of the anti-inflammatory molecule, IL-10 was significantly enhanced in the treated group. In the reversal paradigm **(f**–**j**), the mRNA expression levels of **f** IFN-γ, **g** TNF-α, **h** IL-6, and **i** IL-1β, in the peripheral nerve were significantly reduced by TAK 242 treatment. **j** The mRNA level of IL-10 was unchanged across both groups in the reversal paradigm. *n* = 6/group; *p < 0.05; **p < 0.01; ***p < 0.001

One of the key functions of activated immune cells (macrophages and CD8^+^ T cells) is the release of inflammatory cytokines, which are crucial mediators in neuro-immune interaction. To this end, it became imperative to assess the effect of TLR4 inhibition on the production of inflammatory cytokines. By using quantitative RT-PCR, we measured mRNA levels of major inflammatory cytokines in the sciatic nerve that are implicated in APN. While cytokine expression is generally low or undetectable in intact nerves of healthy WT mice, the mRNA levels of IFNγ (Fig. 5a, f), TNF-α (Fig. 5b, g), IL-6 (Fig. 5c, h), and IL-1β (Fig. 5d, i), were strikingly high in diseased sciatic nerves of L31 mice. In both prevention and reversal paradigms, TAK 242 was able to significantly reduce pro-inflammatory cytokine expression (Fig. 5). Interestingly, in addition to reducing the expression of pro-inflammatory cytokines, TAK 242 strengthened that of anti-inflammatory cytokine, e.g., IL-10 in the prevention paradigm (Fig. 5e). However, such increase was not observed in the reversal paradigm (Fig. 5j). It is interesting to note that in vehicle-treated mice, inflammation at early phase (prevention paradigm, day 21 post-PSNL) was generally much stronger than that at late phase (reversal paradigm, day 38 post-PSNL).

### Myelin and axon integrity in sciatic nerve of L31 mice are preserved following TLR4 signaling blockade

**Fig. 6:**
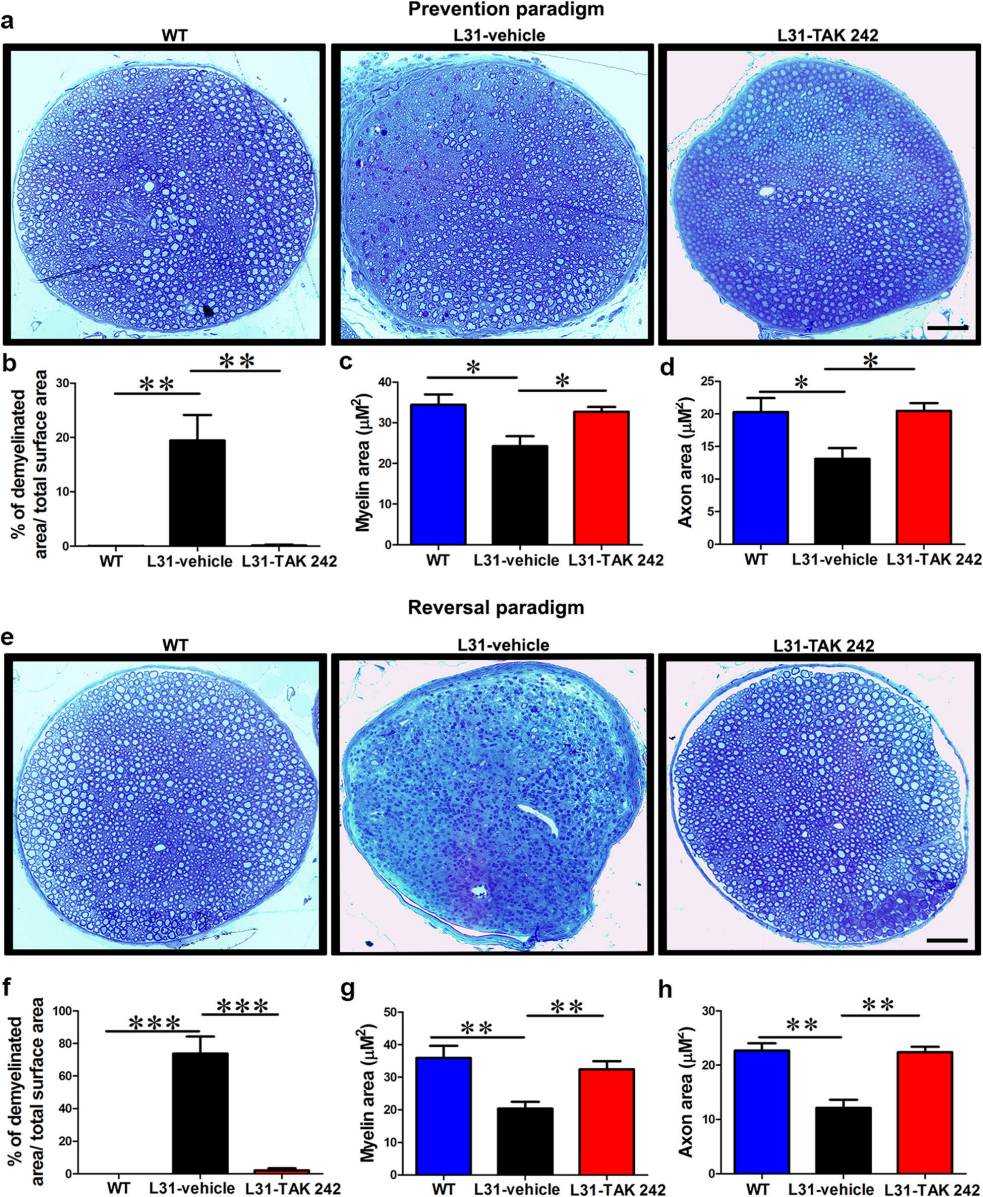
The effect of TLR4 signaling inhibition on axon and myelin integrity in L31-symp mice. In the prevention paradigm (**a**–**d**), **a** representative micrographs of sciatic nerve cross-sections showed partial axonal damage and myelin loss in vehicle group, which was not obvious in TAK 242-treated mice. Nerve structure in TAK 242 remain comparable to WT mice. **b** Semi quantitative analysis revealed a partial axonal and myelin loss (20%) in vehicle-treated mice, which was prevented in TAK 242 mice. **c**, **d** Assessment of myelin and axon area on randomly selected 100 myelinated axons revealed that in the vehicle group, even in the area where nerve structure seems preserved, myelin and axon area was reduced indicating that myelin could become thinner and axons could be atrophic, which were prevented by TAK 242. In the reversal paradigm (**e**–**h**), **e** representative micrographs of sciatic nerve cross sections showed massive demyelination and axonal loss in the vehicle group, TAK 242-treated mice showed an almost intact nerve structure which was comparable to control mice. **f** More than 70% of nerve structure was destroyed in vehicle-treated mice, which was completely rescued in TAK 242-treated mice. **g**, **h** Assessment of myelin and axon area on randomly selected 100 myelinated axons revealed that in the vehicle group, even in the remaining area where nerve structure seems preserved, myelin and axon area was significantly reduced indicating that myelin might become thinner and axons could be atrophic, which were rescued by TAK 242. *n* = 4–6/group; *p < 0.05; **p < 0.01; ***p < 0.001

Demyelination and/or axonal loss are the hallmark pathology in GBS patients and also seen in L31, L31/CD4^−/−^ mice [13]. To confirm our hypothesis that by reducing inflammatory response, TAK 242 can prevent and/or rescue damage in nervous tissue, we performed histological analysis of myelin and axons in sciatic nerves. In WT mice, toluidine staining illustrated a well-organized nerve structure with pale round axons at different sizes. In most cases, axons were surrounded by dark blue myelin rings (Fig. 6a, e). In L31 mice, while tissue samples were collected at day 21 post-PSNL (prevention group), partial damage of the nerve structure (around 20% of the nerve cross-section) was found on the contralateral side of vehicle-treated mice (Fig. 6a, b). When sciatic nerves were harvested at day 38 post-PSNL (reversal group), over 70% of the cross-section of contralateral sciatic nerve of vehicle-treated mice was found severely damaged (Fig. 6e, f). In addition, even in the zone where the nerve structure generally looks maintained, quantitative analysis on randomly selected 100 myelinated axons/section revealed that there was a reduction on both myelin and axon area in vehicle-treated L31 mice at day 21 (Fig. 6a, c, d) as well as day 38 post-PSNL (Fig. 6e, g, h), reflecting a decrease of the myelin thickness and eventually an axonal degeneration. However, TAK 242 treatment starting 2 days prior to PSNL successfully prevented myelin and axonal loss (Fig. 6a, b). Neither severe damage nor micro-injury as assessed by myelin/axon area in relatively well-maintained zone was detected in TAK 242 treated mice (Fig. 6a, c, d). Strikingly, even in the reversal group, TAK 242 was able to completely rescue the nerve damage. It not only prevented further damage starting from the day of the treatment, but also fully rescued already established myelin/axonal loss, resulting in a complete intact nerve structure (Fig. 6), which is in line with functional outcomes described in Fig. 2.

## Discussion

Toll-like receptors (TLRs) are the pattern recognition receptors sensing exogenous and endogenous danger signals. They play important roles in host defense response against intruders by initiating cascades of cytokine and chemokine production. While the involvement of TLRs in other autoimmune disorders including multiple sclerosis [20], systemic lupus erythematosus [21], and autoimmune myocarditis [22] has been well documented, their potential contribution in APN has attracted attention in recent years. By using B7.2 transgenic (L31) mice, an animal model of APN, this study provided clear and convincing evidence that TLR4 is crucial in the initiation and maintenance of inflammatory peripheral neuropathy. Both ligand (HMGB1) and receptor (TLR4) are highly expressed in diseased mice, in the blood and in damaged nerves. Blocking TLR4 signaling with a selective antagonist TAK 242 not only inhibits monocyte, macrophage, and CD8^+^ T cell activation, but also reduces the release of pro-inflammatory cytokines. TAK 242 protects mice from severe demyelination and axonal loss, resulting in a remarkable improvement in mouse motor and sensory function.

TLR4 is most well-known for recognizing lipopolysaccharide (LPS), a component present in many gram negative bacteria. Its ligands also include several viral proteins, polysaccharide, and a variety of endogenous proteins such as low-density lipoprotein, beta-defensins, and heat shock protein [23]. Upon ligand-receptor binding, MyD88 dependent and independent pathways could be activated, leading to the production of pro-inflammatory cytokines. Increased expression of TLR4 in GBS patients have been repeatedly reported [9, 24]. TLR4 299Gly polymorphisms were associated with an increased susceptibility to GBS [25, 26]. While GBS has been considered as a post-infectious neuropathy, a strong response to TLR4 stimulation has been suggested as a critical host condition for *Campylobacter jejuni* triggered GBS [27, 28]. Some pre-clinical studies using EAN as animal model of GBS also revealed TLR4 expression in diseased rats/mice [8, 9]. Similarly, increased expression of HMGB1 have been reported in GBS patients [29, 30]. Serum HMGB1 levels were correlated with disease severity and was reduced following treatment [29]. In our study, an increase of TLR4 and the ligand HMGB1 expression was also detected in L31 mice. Interestingly, the increase in TLR4 expression in the circulating immune cells was initiated before disease onset, which was enhanced when mice became symptomatic, with robust expression of both ligand and receptors in damaged nerves. We have reported previously [15] that L31 and L31/CD4^−/−^ mice have an altered immune background. CD8^+^ T cells in these mice exhibit an effector/memory phenotype, which bears a resemblance to the CD8^+^ T cell response following persistent cytomegalovirus (CMV) infection in humans and mice. Prior exposure to bacterial or viral infection could indeed upregulate TLR4 expression in circulating immune cells, contributing to an altered immune background, which could facilitate the development of GBS.

Similar to many other localized autoimmune disorders where essential pathology is restricted to a specific organ/tissue, in addition to inflammatory damage in peripheral nerve, many GBS patients experience systemic inflammation. The number of neutrophils [31] and monocytes [32] in GBS patients are higher than healthy controls. Blood cytokine levels are found altered, with a significant increase of TNF-α, IL-1β, IL-6, IFN-γ, and C-reactive protein in GBS patients [33, 34]. Systemic inflammation was also detected in EAN animals where TNF-α, IL-6, and IFN-γ serum level parallels disease severity [35, 36]. In our previous study, we also detected a significant increase of various proinflammatory cytokines in the circulation of L31/CD4KO and L31 mice [15], even before the disease onset, where IFNγ and IL-12 could contribute to the generation of CD8^+^ T_EM_ cells by increasing T cell Ags sensitivity [37]. Systemic inflammatory environment is crucial in initiating and maintaining APN. When L31 mice were treated with the TLR4 inhibitor, either prior to or after disease onset, neurological dysfunction and the number of both monocytes and CD8^+^ T cells were significantly reduced, which was associated with an attenuation of their activation status, implying that TLR4 is an important player in systemic inflammation in APN. However, it is worth to pinpoint that, although reduced, TAK 242 was not able to bring the number of circulating immune cells to normal level. It remains significantly higher compared to that of WT mice, even if the nerve structure was restored, and neurological functions were fully recovered. This observation suggests that circulating immune cell expansion is, at least partially, at the upstream of TLR4 signaling and the number of immune cells in the blood might not be a reliable biomarker for nerve pathology.

As a prototypical APN, GBS is characterized by a breakdown of the blood nerve barrier, infiltration of macrophages and lymphocytes to the PNS and demyelination with/or without axonal loss [36, 38]. Nerve macrophages are believed to be effector cells in damaged nerves, which was supported by an increased number of macrophages in GBS patients and in other representative animal models [39–41]. CCL2/CCR2 signaling facilitates the migration of blood derived monocytes to inflamed tissue [42]. Neutralization of CCL2 and 3 inhibited clinical signs of EAN and monocyte recruitment [43]. In this study, we believe that the activation of nerve macrophages, including cell expansion and enhanced TLR4, MHCI expression, was facilitated by the partial ligation that we performed on one of the sciatic nerves, leading to a massive release of endogenous ligands such as HMGB1 for TLR4. Extracellular HMGB1 triggers downstream signaling pathways which have fundamental functions in mediating inflammation, cell migration, proliferation and differentiation [44, 45]. However, a classical nerve injury or DAMPs alone is not sufficient to trigger significant inflammatory response leading to clinically apparent APN, unless the individual possesses a predisposed inflammatory background. L31 mice have an altered immune setting with transgene derived-enhanced B7.2 expression on nerve macrophages, effector/memory CD8^+^ T cells in the blood [15], and increased expression of TLR4 on both monocytes and CD8^+^ T cells in the circulation. When these cells are exposed to DAMPs signals derived from damaged nerves, they exert their effector functions, resulting in inflammatory tissue damage. Furthermore, activated immune cells could also release DAMPs [46], by autocrine and/or paracrine binding to TLRs, they contribute to a vicious cycle of DAMPs release and cytokine production, thus maintaining chronic inflammatory reaction in nerves.

Data obtained from clinical and animal studies suggest the importance of cytokines in the progression and recovery of APN [35, 47]. By using TAK 242, we demonstrated in this study that selective blocking of TLR4 signaling is sufficient to inhibit the inflammatory process in peripheral nerves of L31 mice. Indeed, increased number of macrophages and CD8^+^ T cells as well as proinflammatory cytokines (IFN-γ, TNF-α, IL-1β, and IL-6) in nervous tissue were all attenuated together with an increase of anti-inflammatory cytokine IL-10 in preventive paradigm. Most importantly though, it not only successfully prevented the development of APN, but also fully restored nerve structure integrity and motor/sensory functional in mice with established pathology. It has been documented that in Wallerian degeneration triggered by nerve crush lesion for example, macrophages are required for nerve regeneration. Deficiency in TLR4 signaling impaired macrophage recruitment/activation, resulting in a delayed axonal regeneration and functional recovery. This has been attributed to an insufficient myelin debris clearance due to the lack of macrophage phagocytosis [48, 49]. However, in inflammatory peripheral neuropathy, it appears that macrophages cause myelin and axonal loss predominantly through cytokine storm. Instead of a delay in recovery as seen in Wallerian degeneration, TAK 242 treatment of L31 mice, led to macrophage depletion associated with abolishment of local inflammation, which fully prevented and rescued nerve damage. In L31 mice, macrophage tissue damaging effect was also assisted by effector CD8^+^ T cells, essentially via the release of IFN-γ. With the low expression of Granzyme B, CD8^+^ T cells in the nerves of diseased L31 mice were not considered as classical cytotoxic cells [15].

## Conclusion

**Fig. 7:**
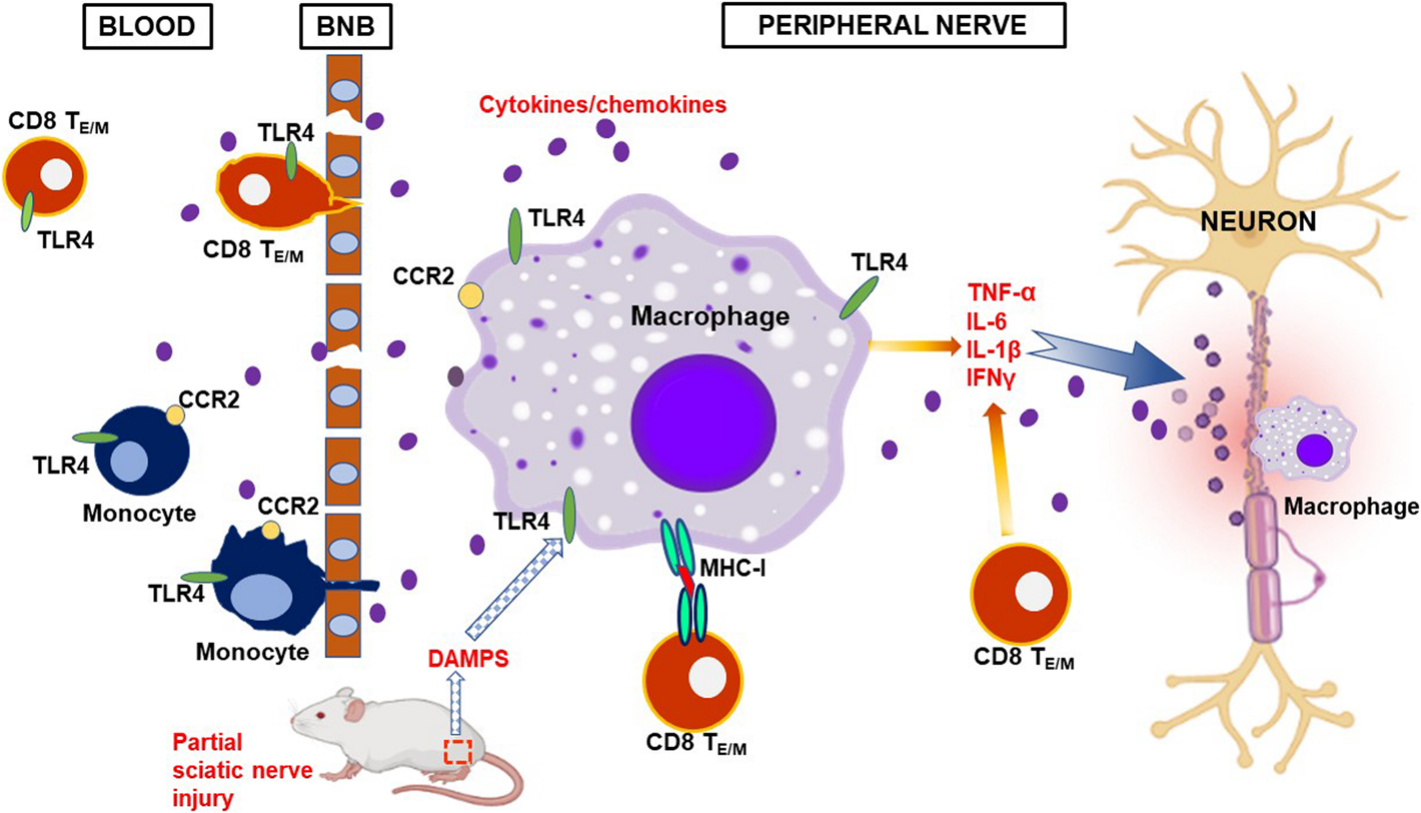
Proposed cascade of TLR4-mediated mechanisms leading to inflammatory peripheral neuropathy in L31 mice. See text for detailed explanation

To sum up, we propose a TLR4-mediated cascade in the development of APN in B7.2 (L31) transgenic mice and its clinical relevance in viral infection triggered GBS. As depicted in Fig. 7, in L31 mice, 1) B7.2 transgene expression increased TLR4 expression in blood monocytes and CD8^+^ T cells in pre-symptomatic mice, together with the presence of effector/memory CD8^+^ T cells, it sets a predisposed immune background for the development of APN; 2) When mice had an injury on one of the sciatic nerves, DAMPs signals derived from damaged nerves further upregulated TLR4 and activated macrophages/CD8^+^ T cells within the PNS; 3) Massive production of proinflammatory cytokines by activated macrophages/CD8^+^ T cells led to demyelination/axonal loss, and neurological dysfunction. Blocking TLR4 signaling effectively abolished immune cell activation and cytokine increase, resulting in protecting nerve structures and improving neurological functions. These findings have significant clinical relevance. Effector/memory CD8^+^ T cells in L31 mice have many resemblances to CD8^+^ T cells following CMV infection which has been listed as one of the most frequent infections triggering GBS. While CMV generates effector/memory CD8^+^ T cells, it also activates TLR4 via PAMPs signaling. When the individual has trauma/surgery, injured nerve can deliver DAMPs signals to further activate TLRs pathway, leading to inflammatory pathology in the PNS. While up to date, the potential involvement of TLR4 in APN was supported mainly by the presence or the upregulation of the receptor and associated molecules in diseased human and animal samples, we demonstrated the causal relationship between TLR4 and the pathogenesis of APN, by using TAK 242, a selective TLR4 inhibitor. The results not only further our understanding on the underlying mechanism of the disease, but also implied TLR4 as a potential target for therapeutic strategy.

## Data Availability

All data are available upon request.

## Abbreviations

APN: Autoimmune peripheral neuropathy
PAMPs: Pathogen-associated molecular patterns
DAMPs: Damage-associated molecular patterns
TLR: Toll-like receptor
GBS: Guillain-Barre syndrome
PNS: Peripheral nervous system
EAN: Experimental allergic neuritis
TIRAP: Toll-interleukin 1 receptor (TIR) adaptor protein
TRIF: TIR-domain-containing adapter-inducing interferon-β
TRAM: TRIF-related adaptor molecule
MyD88: Myeloid differentiation primary response 88
NF-κB: Nuclear factor kappa B
PSNL: Partial sciatic nerve ligation
DMSO: Dimethyl sulfoxide
PFA: Paraformaldehyde
ACK lysing buffer: Ammonium-chloride-potassium lysing buffer
HMGB1: High-mobility group protein 1
qPCR: Quantitative PCR
GAPDH: Glyceraldehyde 3-phosphate dehydrogenase
WT: Wild type
LPS: Lipopolysaccharide
CMV: Cytomegalovirus
MHCI: Major histocompatibility complex I
